# 

**Published:** 1876-02

**Authors:** 


Transactions Medical Society, District of Columbia, Jan., 1876. Pamphlet, pp. 94.

---

Biographical Sketch of John B. Jackson, M.D., of Danville, Ky. By J. M. Toner, M.D., of Washington, D. C., and L. S. McMurtry, M.D., of Danville, Ky. Pamphlet, pp. 11.

---

Tinnitus Aurium, or Noises in the Ears. By Laurence Turnbull, Ph.G., M.D. Pamphlet, pp. 39.

---

Annual Report of Thomas P. Janes, Commissioner of Agriculture of State of Georgia. 1875. Pamphlet, pp. 180.

---

Remarks on Chronic Dysentery. By T. Gaillard Thomas, M.D., Pamphlet, pp. 10.

Wilddungen—Its Baths and Mineral Springs. By Dr. A. Slœcker. Pamphlet, pp. 40.

---

American Association for the Cure of Inebriates. Held in Hartford, Conn. 1875. pp. 98.

